# Effects of green manure planting mode on the quality of Korla fragrant pears (*Pyrus sinkiangensis* Yu)

**DOI:** 10.3389/fpls.2022.1027595

**Published:** 2022-11-29

**Authors:** Sujian Han, Jinfei Zhao, Yang Liu, Linqiao Xi, Jiean Liao, Xinying Liu, Guangdong Su

**Affiliations:** ^1^ College of Mechanical Electrifification Engineering, Tarim University, Alar, China; ^2^ Agricultural Engineering Key Laboratory, Ministry of Higher Education of Xinjiang Uygur Autonomous Region, Tarim University, Alar, China; ^3^ College of Animal Science, Tarim University, Alar, China

**Keywords:** Korla fragrant pear, green manure varieties, fertilizers, fruit quality improvement, comprehensive evaluation model

## Abstract

In this study, a three-year experiment on the fragrant pear orchard was conducted to investigate the effects of different varieties of green manure on the Korla fragrant pear fruit quality, with a view to finding a suitable green manure planting mode for Korla fragrant pear orchard. Green manures were planted in spaces among rows of pear trees, and then smashed and pressed into the soil as fertilisers by the agricultural machinery equipment in their full bloom period. In the experiment, four planting modes of green manure had been set for comparison: SA: Leguminosae green manures alfalfa (*Medicago sativa* L.), SP: Poaceae green manures oats (*Avena sativa* L.), ST: Cruciferae green manures oilseed rape (*Brassica napus* L.), and S: orchard authigenic green manures (*Chenopodium album* L., *Mulgedium tataricum* (L) DC., and *Phragmites australis* (Cav.) Trin. ex Steud.). Apart from that, eleven fruit quality indicators were analyzed to evaluating the effects of different green manure planting mode on the quality of fragrant pear. According to analysis of variance (ANOVA) results, there were significant differences among four planting modes in terms of nine fruit quality indicators (*P*<0.05). In addition, the correlation analysis (CA) results revealed that there were different degrees of correlations among quality indicators. On this basis, repeated information among indicators was eliminated by principal component analysis (PCA), thus simplifying and recombining the three principal components. All in all, these three principal components reflect appearance traits, internal nutritive value and taste of fruits, respectively. Specifically, SA significantly improved the internal quality and nutritive value of fruits, SP improved the physical traits of fruits, and ST significantly improved the taste of fruits. Based on the PCA results, a comprehensive evaluation model of fruit quality was constructed. The are comprehensive fruit quality scores:SA>SP>ST>S.

## 1 Introduction

Korla fragrant pear (*Pyrus sinkiangensis* Yu), which has been planted for over 1,300 years, is not only a special high-quality fragrant pear species in Xinjiang, China, but also a geographical indicator of agricultural products in China (Cheng et al., 2021; Wang et al., 2021a). At the same time, it is popular among consumers due to its distinct appearance, jade-like colours, high sugar content and high nutritive value. Moreover, Korla fragrant pear has been exported to many countries and regions around the world (Simmonds and Preedy, 2015; Yu et al., 2021). Nutrient supplementation in fragrant pear orchards is mainly dependent on chemical fertilisers. It is noteworthy that excessive application of fertilisers may lead to poor fruit quality traits (Zhao et al., 2017) and failure to meet standards for ‘green organic fruits’ (Vega-Muñoz et al., 2022). Beyond that, poor orchard management mode significantly restricts the continuous health development of the fragrant pear industry. Hence, exploring a new clean fertiliser source in orchards that can improve fruit quality and reduce reducing chemical fertiliser consumption is conducive to enhancing the brand reputation and economic benefits of Korla fragrant pears.

Using green cover crops as plant fertility is an effective environmental protection measure to improve fruit quality and decrease fertiliser consumption (Srivastava et al., 2007; Chen et al., 2014; Dong et al., 2021). Meanwhile, some scholars have studied the effects of green manures on pear orchards. For example, Lee et al. (2014) compared the influences of *Astragalus sinicus* and rye on the fruit weight, soluble solids and titratable acids of fragrant pear fruits. In addition, the co-planting of *Astragalus sinicus* and *Lathyrus cicera* providing nutrients for the growth of pear trees while improving the tourism and ecological functions of pear orchards (Zhang et al., 2021a). Leguminosae, Poaceae and Cruciferae are common types of green manures in fruit orchards (Yim et al., 2017; Deakin et al., 2018; Zhong et al., 2018). It is found that the soil management mode of planting leguminous green manure (*Vicia faba* L.) and burying cutting residues from the main crop could increases grape output and soluble solid content effectively (Pisciotta et al., 2021). As demonstrated by Zhu et al. (2022), Poaceae green manure could increase the total nutritional value and fruit quality in wolfberry orchard, with significant increases in carotenoids and Vitamin C contents. Oilseed rape is a common Cruciferae green manure in agricultural production (Gao et al., 2022). According to Wang et al. (2020), using high-concentration rapeseed residues as fertiliser increases sugar content (total soluble sugar and water-soluble sugars) significantly. To sum up, planting green manures in orchards could improve the quality of fruits, and different varieties of green manures have varying degrees of influence on fruit quality. However, there has been limited research on the benefits of different planting mode of green manure on fruit quality enhancement in Korla fragrant pear orchards.

The soil environment such as temperature, humidity and microflora in an orchard is relatively complicated. It is difficult to calculate and predict the release and utilisation of elements in green manure crops accurately (Rodrigues et al., 2013). The effects of green manures in orchards can be reflected by comparing improvement of fruit quality (Ambrosano et al., 2018). Principal component analysis (PCA), which has been extensively applied, can positively affect the comprehensive assessment of fruit quality, scientifically and objectively reflect the correlation of quality indicators of fruits, and simplify recombination analysis (Milošević et al., 2022; Song et al., 2022). In addition, Cozzolino et al. (2020) identified indicators related to volatile matters, smells and tastes of six kiwi fruit varieties by PCA, and PCA could classify kiwifruit varieties based on quality indicators. Furthermore, Zheng et al. (2022) recombined 15 quality indicators of nine pear varieties in North China into four principal components using PCA, established a comprehensive evaluation model of pear varieties and provided theoretical references to the cultivating and screening of fragrant pear varieties in North China. To sum up, PCA can simplifies fruit quality indicators reasonably through dimension reduction, solve the inexplicit boundary between primary and secondary quality indicators of fruits, and lay the foundations for the quality assessment system and scientific classification of fruits.

In this study, Four planting modes of green manure were set in Korla pear orchard. Moreover, eleven fruit quality indicators were chosen for data processing through the analysis of variance, correlation analysis and principal component analysis. On this basis, a comprehensive evaluation model of fruit quality was constructed to finding a suitable green manure planting mode for Korla fragrant pear orchard. In short, the research results provide some theoretical references to the green manure type and planting mode in Korla fragrant pear orchards in Xinjiang.

## 2 Materials and methods

### 2.1 Experimental design

The experimental site is located in the modern organic fragrant pear demonstration base in Twelve group, Alar City, Xinjiang Uygur Autonomous Region (81°26′E, 40°28′N). It belongs to a warm temperate desert climate and the soil type is sandy loam. The annual average solar radiation is 133.7~146.3 kilocalorie/cm^2,^ and the annual average sunshine is 2556.3~2991.8 h. There is rare precipitation in Xinjiang, with an annual average precipitation of only 40.1~82.5 mm. Moreover, there is strong surface evaporation, and the annual average evaporation capacity is 1876.6~2558.9 mm.

Six-year-old Korla fragrant pear trees (until 2021) were chosen as the test samples. The rootstock used was the three-year *Pyrus betulaefolia*, and Dangshan Pear was used as the pollination variety. The space between pear tree rows was 4 m × 6 m, and the average tree height was 3.5 m. Artificial cultivation of green manure and natural grass growth were adopted in spaces between the rows of the orchard. A total of four groups were set: SA: Leguminosae green manure alfalfa (*Medicago sativa* L.), SP: Poaceae green manures oats (*Avena sativa* L.), ST: Cruciferae green manures oilseed rape (*Brassica napus* L.), and S: orchard authigenic green manures (authigene grass). In the orchard, authigenic green manures were mainly *Chenopodium album* L., *Mulgedium tataricum* (L) DC., and *Phragmites australis* (Cav.) Trin. ex Steud. Each group had three repeated blocks, which were arranged randomly. Each block contained 24 pear trees. The pear trees were arranged in 3 rows × 8 columns. Green manures were planted in spaces between rows of pear trees.

Green manure planting commenced on April 2019. Before planting the green manure, the experimental blocks were deep ploughed with farm machinery equipment. The seeds of green manure were sown by machines in lines in the SA, ST and SP blocks on the first 10 days of April every year. The sowing amount of Alfalfa seeds was 9.75 kg/ha, and the sowing depth was 1.5 cm. The sowing amount of oat seeds was 19.5 kg/ha, and the sowing depth was 3 cm. The sowing amount of rape seeds was 15 kg/ha, and the sowing depth was 2 cm. Three flood irrigation were provided to each block every year in the last 10 days of April, the first 10 days of June and the last 10 days of July, respectively. In the middle 10 days of each month, SA, SP, and ST blocks were manually weeded, while weeds in S blocks remained. In the first 10 days of July (the full bloom stage of green manure), green manures and authigene in all blocks were smashed and pressed into soils using agricultural machinery equipment (the press-in depth of smashed green manures was about 15 cm). Water and fertiliser management of trees, pruning, flower and fruit management, disease and pest control, and other technological requirements during the planting process of all test blocks referred to the implementation of technical regulations for Korla fragrant pear production (Ministry of Agriculture of the People's Republic of China, 2004). The details of regulation are as follows: 1. Soil management: Combined with the use of base fertilizer in autumn for deep turning, before the winter for flood irrigation. 2. Water supplement: According to the need of fruit trees and soil conditions reasonable irrigation. 3. Flower and fruit management: Fine pruning, artificial pollination, and bee release in pear orchard; Flower and fruit thinning, control the load of single plant. When there are few flowers and fruits, pay attention to protecting flowers and fruits. 4. Pest control: Pay attention to the protection and utilization of natural enemies, maintain the ecological balance of farmland, reduce environmental pollution.

### 2.2 Fruit collection and indicator test methods

#### 2.2.1 Fruit harvest

On 26 September 2021, 150 fruits were harvested for each of the four treatments. Tree selection and fruit harvesting were introduced as follows: 15 healthy pear trees with constant growth conditions and no diseases or pests were chosen for each group. The pear trees were planted with the same green manure on both sides. Ten fruits were harvested from the east, south, west, north, and top positions of each tree. The fruits’ weights, transverse diameters, and vertical diameters were measured after they were packed in plastic foam net bags and brought back to the laboratory. Next, the fruits were stored in a refrigerator (temperature: 0 ± 0.5 °C; relative humidity: 90 ± 5%). On the second day, fruits were taken out from the refrigerator to test the residual indicators.

#### 2.2.2 Selected test methods for fruit indicators

A total of 11 quality indicators related to appearance traits, internal quality, nutritive value, and tastes of fragrant pear fruits were chosen. Specifically, single-fruit weight, transverse diameter, vertical diameter, and shape index reflect the appearance traits of fruits. Soluble solid and fruit hardness reflect the internal quality of the fruits. The content of Vitamin C and protein content reflects the nutritive value of fruits. Titratable acid, total soluble sugar, and sugar-acid ratio reflect the tastes of fruits. The single-fruit weight, transverse diameter, vertical diameter, Soluble solid content, Fruit hardness and shape index of each treatment were measured using 150 fruits. In addition, 150 fruits from each treatment were divided into 50 groups (3 pears per group). 30 g of pulps from each pear in the group was mixed and used for measurement of vitamin C, protein, Total soluble sugar, and titratable acid. The measurement methods of different indicators are introduced as follows:

Single-fruit weight (SFW): dust was removed from the fruit’s surface before the fruit was weighed on an electronic scale. After the numerical value of the electronic scales was stabilised, data were recorded, and the mean of the two measurements was selected (unit: g).

Transverse diameter of fruits (TD): the diameter of the fruit’s bellies was measured at every 180° angle by an electronic vernier caliper. The mean of the two measurements was selected (unit: mm).

Vertical diameter of fruits (VD): distance from the fruit stem to the bottom was measured by an electronic vernier caliper. The mean of the two measurements was selected (unit: mm).

Shape index (SI): vertical diameter/transverse diameter.

Fruit hardness with skin (FH): three points at the equator of fragrant pear (interval: 120°) were selected, and peak mode was chosen using the GY-4 fruit hardness metre. The indenter was pressed vertically into fragrant pear into 10 mm, and data were recorded (unit: kg/cm^2^).

Soluble solid content (SSC): soluble solid was tested by a PAL digital display sugar metre. After eliminating pericarps and kernels from pear fruits, the pulps were mixed uniformly, in which 5 g of pulps were wrapped in gauze to extract 1 ml of juice. The juice was dripped on the endoscope of the sugar displayer to read the numerical values (unit: %). Each group had five repetitions, and the mean values were collected.

Vitamin C (VC): the VC content was tested by the molybdenum blue colorimetric method, which was changed slightly according to Sayed and Soliman’s, 1979) method. In a mortar, 4 g of fresh pulp was combined with 5 mL oxalic acid-EDTA solution before being ground into a homogenate. After transferring the homogenate to a centrifuge tube, 5 mL of the oxalic acid-EDTA solution was added to the mortar to rinse it. The solution was then transferred into a centrifuge tube. The centrifuge tube was centrifuged for 20 minute at the rate of 4500 r/min, and then 1 mL of supernate was transferred into the test tube. Afterwards, 4 mL of oxalic acid-EDTA solution, 0.5 mL of metaphosphoric acid-acetic acid, 1 mL of 5% sulfuric acid solution and 2 mL of 5% ammonium molybdate solution were added. The liquid in the test tube was vibrated uniformly and then put in a kettle for 15 minute of water bath under 30 °C. Colourimetry was performed under a wavelength of 760 nm, and absorbance was recorded (unit: mg/100 g). Each sample was repeated three times, and the mean values were chosen.

Protein content (PRO): the Coomassie brilliant blue (CBB) G-250 staining method was used (Bradford, 1976). Fresh pulp (1 g) was put in a mortar, and 5 mL of distilled water was added to grind into the homogenate. The homogenate was transferred into a centrifuging tube. Next, 5 mL of distilled water was used to rinse the mortar. In the centrifuge tube, the solution was mixed. The centrifuging tube was put in an ultrasonic wave oscillator for 15 minute to mix evenly. Afterwards, it was centrifuged at 5500 r/min for 10 minute. Subsequently, 0.5 mL of supernatant was transferred to a test tube along with 0.5 mL of distilled water and 5 mL CBB reagent; they were thoroughly combined, and the absorbance of soluble proteins was recorded at 595 nm (unit: g/100 g). Each sample was replicated three times, and the means were chosen.

Total soluble sugar (TSS): tested by anthranone-sulfuric acid colourimetry with references to the method of Laurentin and Edwards (2003). Fresh pulp (1 g) was put in a mortar, and 5 mL of distilled water was added to produce a homogeneous mixture. The homogenate was transferred into a centrifuging tube. Then, 5 mL of distilled water was used to rinse the mortar.The solution was mixed into the centrifuge tube, which was then placed in a water bath kettle and heated for 30 minute at a temperature 80 °C. The grinding fluid was cooled and centrifuged for 10 min at 4000 r/min. The supernatant was transferred to a volumetric flask. Subsequently, 10 mL of distilled water was added to the sediments in the centrifuge tube. The water bath and centrifugation processes were repeated twice until the supernatant in the volumetric flask was dissolved to a constant volume of 25 mL. Subsequently, 1 mL of solution was transferred into a volumetric flask and dissolved into 100 mL (by 100 times) distilled water. Later, 2 mL of the collected solution was combined with 5 mL of the anthranone-sulfuric acid reagent using vibration. The mixture was heated with boiled water for 10 minute. After the mixture was cooled, the absorbance was determined at 620 nm (unit: %). Each sample was repeated three times, and the mean values were determined.

Titratable acid (TA): the acid-base titration method was applied (Liu et al., 2009). Fresh pulp (1 g) was put in a mortar, and 5 mL of distilled water was added to grind into homogenate. The homogenate was transferred into a centrifuging tube. The mortar was then rinsed using 5 mL of distilled water. The solution was added to the centrifuging tube, which was then placed in a water bath kettle and heated at 80 °C for 30 minute. The grinding fluid was cooled and centrifuged for 10 minute at 4000 r/min. The supernatant was transferred to a volumetric flask. Next, distilled water was added to the sediments in the centrifuging tube. The above water bath and centrifugation processes were repeated twice until the supernatant in the volumetric flask was dissolved to a constant volume of 25 mL. Later, 20 mL of the solution was collected and titrated with NaOH solution (0.1 mol L^-1^) until pH = 8.0. Titratable acid content was calculated according to the titration volume of NaOH solution (unit: percentage of malic acid (%)). Each sample was repeated three times, and the mean values were determined.

Sugar-acid ratio (SAR) refers to the ratio between the total soluble sugar and titratable acid of fruits.

### 2.3 Devices and instruments

In this study, the following devices and instruments were utilised: Japan ATAGO fruit sugar metre PAL-1 (Guangzhou Atang Scientific Instrument Co., LTD), GY-4 fruit hardness metre (Yueqing Aidebao Instrument Co. LTD), ultrapure water metre UPT-1-107 (Europtronic Group), ThermoFisher Biomate 160 ultraviolet and visible spectrophotometer (ThermoFisher Scientific China Co., LTD), KQ5200E ultrasonic cleaner (Kunshan Ultrasonic Instrument Co., LTD), American thermoelectricity Sorvall ST16R high-speed centrifuge (ThermoFisher Scientific China Co., LTD), DK-8D digital display water bath kettle (Jintan City Medical Instrument Factory), and FA1104 electronic scales (Shanghai Jinghua Technology Instrument Co., LTD).

### 2.4 Data processing and diagram plotting

Firstly, the method of analysis of variance was determined according to whether the fruit quality indicators data conform to univariate normal distribution. The Kolmogorov-Smirnov (Barrera et al., 2021) statistical method in SPSS software (Version 25.0 IBM, USA) was used to analyze the single factor normality of fruit quality indexes. When “Sig” value is less than 0.05, the original fruit indicator data is considered to be non-normal distribution. As shown in **Table 1**, among the eleven fruit quality indicators, only vertical diameter of fruit (VD) conforms to the normal distribution. Therefore, based on the method of Kruskal-Wall nonparametric multiple comparison (all pairwise) (Wang et al., 2022), one-way analysis of variance (K sample) was used in SPSS software to compare the significant differences between the medians of fruit quality indicators under different green manure planting modes (*P*<0.05).

**Table 1:** Kolmogorov-Smirnov univariate normality test for fruit quality indicators.

Fruit quality index	Most Extreme Differences			AsymptoticSig
	Absolute	Positive	Negative	
SFW/(g)	0.061	0.061	-0.036	&
TD/(mm)	0.067	0.067	-0.052	&
VD/(mm)	0.036	0.036	-0.034	0.06
SI	0.08	0.08	-0.034	&
SSC/(%)	0.155	0.155	-0.083	&
FH/(kg/cm²)	0.158	0.133	-0.158	&
VC/(mg/100g)	0.077	0.077	-0.052	0.006
PRO/(g/100g)	0.067	0.052	-0.067	0.029
TSS/(%)	0.117	0.117	-0.079	&
TA/(%)	0.076	0.047	-0.076	0.007
SAR	0.127	0.127	-0.089	&

The symbol “&” in the table represents the “Sig” value of fruit index is far smaller than 0.05.

**Figure 1:**
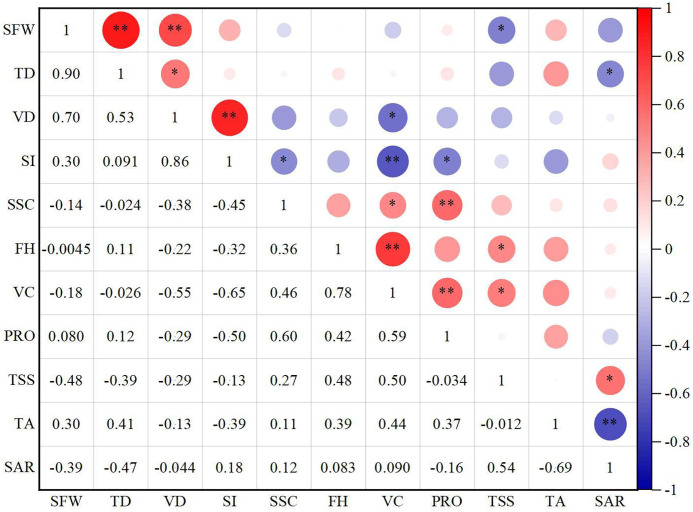
Spearman correlation analysis of fruit quality indicators of fragrant pear. Red and blue represent the positive and negative correlations among the quality indicators. The darker colour represents the stronger significance. ** refers to the 0.01 level, which indicates a significant correlation. * refers to the 0.05 level, which indicates a significant correlation. SFW, TD, VD, SI, SSC, FH, VC, PRO, TSS, TA, and SAR represent abbreviations of single-fruit weight, transverse diameter, vertical diameter, shape index, soluble solid content, fruit hardness, vitamin C, protein, total soluble sugar, titratable acid, and sugar-acid ratio respectively. The numbers in the left half of the figure represent the correlation coefficients between indicators (r).

**Figure 2:**
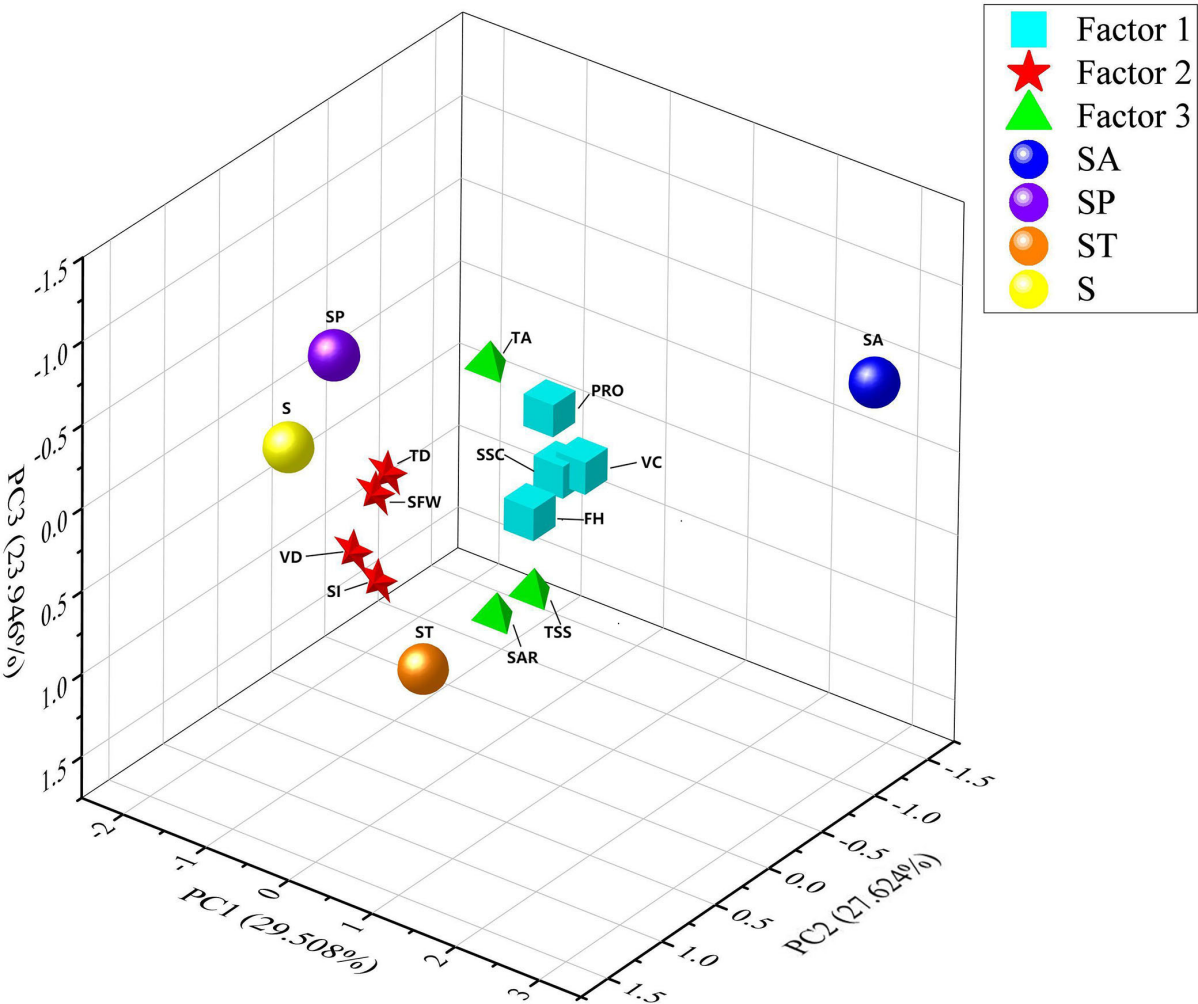
Three-dimensional scattering point diagram of factor composition and sample scores. The cube, star and tetrahedron represent PC1, PC2 and PC3, respectively.

Secondly, the correlation analysis (CA) method was selected according to whether the fruit quality indicators obey the bivariate normality. The bivariate normality between fruit quality indicators was tested based on Doornik-Hansen multivariate normal analysis method (Doornik and Hansen, 2008) in Stata software (Version 17). As shown in **Table 2**, when “Prob-chi2” value is greater than 0.05, it is considered that the two fruit indicators conform to the bivariate normal distribution, and the results show that all fruit quality indicators do not obey the bivariate normality. Therefore, Spearman’s method was adopted in study to analyze the correlation between fruit quality indicators (Li et al., 2021). In addition, SPSS software was used for normalisation of the original fruit indicators data and principal component analysis (PCA), and Origin software (version 2021) was used to draw the CA diagram (**Figure 1**) and three-dimensional scattered point diagram (**Figure 2**). As shown in **Table 3**, in order to more comprehensively show the effects of different green manure planting modes on fruit quality, the results of each indicator are expressed as mean ± standard deviation. Different letters after the mean indicate significant differences at the 0.05 level between the medians of fruit quality indicators for different treatments (**Table 4**).

**Table 2:** Doornik-Hansen bivariate normality test for fruit quality indicators.

Pair of variables			Prob-chi2	Pair of variables		Prob-chi2	Pair of variables		Prob-chi2
SFW	TD	&		VD	SI	0.006	SSC	TA	&
SFW	VD	&		VD	SSC	0.001	SSC	SAR	&
SFW	SI	&		VD	FH	&	FH	VC	&
SFW	SSC	&		VD	VC	&	FH	PRO	&
SFW	FH	&		VD	PRO	0.01	FH	TSS	&
SFW	VC	&		VD	TSS	&	FH	TA	&
SFW	PRO	0.001		VD	TA	0.003	FH	SAR	&
SFW	TSS	&		VD	SAR	0.001	VC	PRO	&
SFW	TA	&		SI	SSC	&	VC	TSS	&
SFW	SAR	&		SI	FH	&	VC	TA	&
TD	VD	0.031		SI	VC	&	VC	SAR	&
TD	SI	&		SI	PRO	0.001	PRO	TSS	0.001
TD	SSC	&		SI	TSS	&	PRO	TA	0.007
TD	FH	&		SI	TA	0.002	PRO	SAR	0.023
TD	VC	&		SI	SAR	0.002	TSS	TA	&
TD	PRO	0.005		SSC	FH	&	TSS	SAR	&
TD	TSS	&		SSC	VC	&	TA	SAR	&
TD	TA	0.003		SSC	PRO	&			
TD	SAR	&		SSC	TSS	&			

The symbol “&” in the table represents the “Prob-chi2” value of fruit index is far smaller than 0.05.

**Table 3:** Significance analysis of fruit quality indicators under four planting modes of green manures.

Fruit quality index	SA	SP	ST	S	Average value	Standard Deviation	Coefficient of Variation%
SFW/(g)	163.81 ± 13.2(a)	183.26 ± 21.5(a)	164.3 ± 4.93(a)	170.5 ± 11.91(a)	170.46	15.32	8.99
TD/(mm)	65.9 ± 1.7(b)	68.07 ± 1.83(a)	65.66 ± 0.51(b)	66.37 ± 1.51(ab)	66.50	1.67	2.50
VD/(mm)	67.32 ± 2.54 (b)	71.36 ± 4.69(a)	70.6 ± 2.13(a)	71.26 ± 1.94(a)	70.13	3.26	4.64
SI	1.02 ± 0.02(a)	1.05 ± 0.04(a)	1.07 ± 0.04(a)	1.07 ± 0.02(a)	1.05	0.04	3.41
SSC/(%)	14.08 ± 0.59(a)	12.56 ± 0.2(c)	12.78 ± 0.26(b)	12.85 ± 0.67(b)	13.07	0.75	5.72
FH/(kg/cm²)	13.66 ± 0.5(a)	13.07 ± 0.33(b)	11.52 ± 1.49(c)	10.43 ± 0.92(d)	12.17	1.56	12.80
VC/(mg/100g)	6.64 ± 0.66(a)	5.57 ± 0.18(b)	5.18 ± 0.25(c)	4.61 ± 0.21(d)	5.51	0.83	15.12
PRO/(g/100g)	0.25 ± 0.01(a)	0.23 ± 0.01(b)	0.22 ± 0.01(b)	0.22 ± 0.02(b)	0.23	0.02	8.26
TSS/(%)	14.21 ± 0.81(a)	12.94 ± 1.64(b)	13.23 ± 1.66(b)	12.66 ± 0.28(b)	13.26	1.29	9.73
TA/(%)	0.081 ± 0.003(b)	0.088 ± 0.007(a)	0.067 ± 0.008(c)	0.078 ± 0.005(b)	0.078	0.01	12.21
SAR	174.9 ± 5.4(b)	149.69 ± 30.3(d)	199.66 ± 33.69 (a)	163.55 ± 14.34 (c)	172.90	29.55	17.09

SFW, TD, VD, SI, SSC, FH, VC, PRO, TSS, TA, and SAR represent abbreviations of single-fruit weight, transverse diameter, vertical diameter, shape index, soluble solid content, fruit hardness, vitamin C, protein, total soluble sugar, titratable acid, and sugar-acid ratio respectively. The results of all indicators are expressed as mean ± standard deviation. After averaging the values of fruit quality indicators in the same row, different letters indicate significant differences at the 0.05 level.

**Table 4:** Full name and Abbreviations.

Full name	Abbreviations	Full name	Abbreviations
Analysis of variance	ANOVA	Single-fruit weigh	SFW
Correlation analysis	CA	Transverse diameter	TD
Coefficient of variation	CV	Vertical diameter	VD
Leguminosae green manure alfalfa (*Medicago sativa* L.)	SA	Shape index	SI
Poaceae green manures oats (*Avena sativa* L.)	SP	Soluble solid content	SSC
Cruciferae green manures oilseed rape (*Brassica napus* L.)	ST	Fruit hardness	FH
Orchard authigenic green manures (antigenic grass)	S	Vitamin C	VC
Principal component analysis	PCA	Protein	PRO
Principal component 1	PC1	Total soluble sugar	TSS
Principal component 2	PC2	Titratable acid	TA
Principal component 3	PC3	Sugar-acid ratio	SAR

## 3 Results and analysis

### 3.1 ANOVA of fruit quality

**Table 3** shows the results of quality indicators of fragrant pears under different green manure modes. Among 11 quality indicators, there were significant differences in ANOVA results for nine indicators (*P*<0.05), indicating that different green manures can influence fruit quality to some extent. With respect to the appearance traits, fruit weight and appearance are not only important business characteristics, but also the key determinants of customer selection (Isuzugawa et al., 2014; Lyu et al., 2017). There was no significant (*P*>0.05) difference between the four treatments in terms of fruit weight, and all groups met the standards of special-grade fruits in the Korla fragrant pear industrial evaluation (SFW>160 g). In addition, Fruit shape (SI) is also an important commercial feature of fruit. Consumers prefer the oval shaped pears. In general, the smaller the value of SI, accompanied by rounder fruit shapes. There was no significant difference in fruit shape among the four treatments in this study, indicating that different varieties of green manure have little effect on fruit shape. In general, substances that dissolve in water are referred to as soluble solids, and they are made up of sugar, acid, vitamins and minerals. SSC is a major indicator for measuring the maturity of Korla fragrant pear (Niu et al., 2020). Among the four groups, the SSC of SA was significantly higher than that of the other three groups (*P*<0.05) (**Table 3**). Apart from that, the fragrant pears with higher hardness have better storage performances and stronger resistance to damage during transportation (Yu et al., 2018). Hardness is an important indicator that influences the taste of fruits. In this study, SA and SP substantially improved the hardness of fragrant pears. VC, also known as ascorbic acid, is an indispensable nutrient that maintains normal human physiological functions. It is worth mentioning that the human body cannot synthesise VC independently, but can only acquire it from foods. Hence, VC is an important indicator to measure the nutritive value of fruits (Wang et al., 2021b). There were significant differences between the VC content of the four groups of fruits (*P*<0.05). Specifically, SA had the highest VC content (**Table 3**), while S had the lowest. Moreover, protein is an important bearer of vital human activities. Compared to animal proteins, plant proteins are more easily absorbed by the human body and contain more nutrients. It is also a significant indicator to measure the nutritive value of fruits (Liu et al., 2014; Zhang et al., 2021b). The fruit protein content of SA was significantly higher than that of other groups (*P*<0.05). Therefore, this study demonstrated that SA could substantially improve the nutrient content of fruits. SAR is a common indicator that evaluates the taste and flavour of fruits. Unlike other pear varieties, Korla fragrant pear has higher SAR (Chen et al., 2007; Aprea et al., 2017). SAR is determined by soluble sugar and titratable acid. The results showed that the TSS of SA was significantly higher than other three groups (*P*<0.05), and there were significant differences in terms of TA content (*P*<0.05): SP>SA>S>ST (**Table 3**). Among the 11 indicators, SAR presented the highest Coefficient of Variation (CV), reaching 17.09%. Indicating that green manure has the most significant influence on the SAR of fruits. In general, SP contributed the highest SFW, SA presented the highest nutritive value of fruits, and ST achieved the highest SAR of fruits.

### 3.2 CA of fruit quality indicators

The correlation coefficients (r) of quality indicators of fragrant pears were shown in **Figure 1**. SFW had extremely significant positive correlations with TD and VD of fruits (*P*<0.01). There was an extremely significant positive correlation between VD and SI of fruits (*P*<0.01). A highly significant positive connection exists between FH and VC (*P*<0.01). The PRO of fruits revealed substantial positive correlations with SSC and VC (*P*<0.01). SI had extremely significant negative correlations with VC (*P*<0.01). TA had highly significant negative correlations with SAR (*P*<0.01). There was a significant positive correlation between TD and VD of fruits (*P*<0.05). SSC had significant correlations with VC (*P*<0.05). The TSS of fruits had significantly positive correlations with FH, VC, and SAR (*P*<0.05). In addition, SFW had significantly negative correlations with TSS (*P*<0.05). A significant negative connection exists between TD and SAR (*P*<0.05). There was a significant negative correlation between VD and VC of fruits (*P*<0.05). The SI of fruits revealed significant negative correlations with SSC and PRO (*P*<0.05). In summary, indicators of fruits influence one another rather than being entirely independent, exhibiting varying degrees of positive or negative correlations. For a more thorough assessment of fruit quality, it was necessary to separate out information that was repeated among indicators, perform a streamlined recombination and analyse every indicator.

### 3.3 PCA of fruit quality indicators

PCA can be used to recognise potential trait combinations among fruit quality indicators. The PCA results of fruit quality indicators were listed in **Table 5**. Three common factors (PC1, PC2 and PC3) with characteristic roots higher than one were extracted, and their contribution rate to the total variance reached 81.078%. They were sufficient to interpret the majority of pear fruit quality parameters. The contribution rate of PC1 was 29.508%. PC1 was composed of VC, FH, SSC and PRO of fruits, reflecting the internal quality and nutritive value of fruits. PC2 consisted of SFW, TD, VD and SI, with a contribution rate to a total variance of 27.624%. It mainly reflects the appearance traits of fruits. The contribution rate of PC3 to the total variance was 23.946%. PC3 was composed of TSS, TA and SAR, reflecting the saccharic acid content of fruits.

**Table 5:** Post-rotating principal component vector matrix and total variance interpretation.

Quality index	Common Factor 1	Common Factor 2	Common Factor 3
SFW	-0.023	0.949	-0.231
TD	0.052	0.896	-0.345
VD	-0.347	0.898	0.204
SI	-0.482	0.564	0.551
SSC	0.733	-0.169	-0.036
FH	0.829	0.179	0.05
VC	0.916	-0.234	-0.068
PRO	0.595	-0.178	-0.375
TSS	0.582	-0.064	0.675
TA	0.363	0.197	-0.761
SAR	0.032	-0.148	0.966
Characteristic root	3.246	3.039	2.634
Factor contribution rate %	29.508	27.624	23.946
Cumulative variance contrition rate %	29.508	57.132	81.078

SFW, TD, VD, SI, SSC, FH, VC, PRO, TSS, TA, and SAR represent abbreviations of single-fruit weight, transverse diameter, vertical diameter, shape index, soluble solid content, fruit hardness, vitamin C, protein, total soluble sugar, titratable acid, and sugar-acid ratio respectively.

The spatial component diagram of PCA and the scattering point diagram of fragrant pear fruit scores under the four green manure types were fitted (**Figure 2**). Spheres in different colours represent the spatial coordinate points of fruit indicator scores under four green manure types. Scores of fragrant pear samples on PC1, PC2 and PC3 were calculated according to the following formula:


$$f_{1}=-0.01q_{1}+0.03q_{2}-0.19q_{3}-0.27q_{4}+0.41q_{5}+0.46q_{6}+0.51q_{7}+0.33q_{8}+0.32q_{9}+0.20q_{10}+0.02q_{11}$$

$$f_{2}=0.54q_{1}+0.51q_{2}+0.52q_{3}+0.32q_{4}-0.10q_{5}+0.10q_{6}-0.13q_{7}-0.10q_{8}-0.04q_{9}+0.11q_{10}-0.08q_{11}$$

$$f_{3}=-0.14q_{1}-0.21q_{2}+0.13q_{3}+0.34q_{4}-0.02q_{5}+0.03q_{6}-0.04q_{7}-0.23q_{8}+0.42q_{9}-0.47q_{10}+0.60q_{11}$$

where *f_1_, f_2_*, and *f_3_* were scores of fragrant pear samples on three coordinate axes of PC1, PC2 and PC3. The values of *q_1_* ~*q_11_* represent the original data of pear fruit indexes after normalisation by SPSS software. Coefficients in front of *q_1_* ~*q_11_* were calculated as follows.


$$\text{Factor Eigenvector Coefficient}=\frac{\text{Factor Load}}{\sqrt{\text{Corresponding factor characteristic root}}}$$

where Factor Load reers to vector vales corresponding to 11 quality indicators (SFW, TD, VD, SI, SSC, FH, VC, PRO, TSS, TA and SAR) in **Table 5** from common factor 1 to common factor 3.

In **Figure 2**, four indicators of PC1 (VC, PRO, SSC and FH) and four indicators of PC2 (SFW, TD, VD and SI) presented relatively dense spatial distributions. In other words, there were close connections and repeated information among the indicators. The results agree with CA in **Figure 1**. There was scattered spatial distribution in PC3 because TA was reverse loads and negatively affected PC3 and fruit quality. This conformed to the practical conditions of fruit evaluation of pears.

The dimensions of the chosen 11 indicators were reduced, and they were recombined into three main traits through PCA. These three traits reflected appearance, internal nutritive value and tastes. Overlapping information among indicators was eliminated well, and the scale of indicators was shrunk. Spatial distributions of spheres in different colours in the three-dimensional diagram intuitively reflect the influences of different green manure types on the quality traits of fruits. SA received the highest score on PC1. Compared with other treatments, SA significantly improved the internal quality and nutritive value of the fruits. SA achieved the highest scores in the four indicators of PC1. Moreover, SA achieved the lowest scores on PC2, indicating that SA slightly improved the fruit weight and shape of fragrant pears. This conformed to the ANOVA results. SP achieved the highest scores on PC2. Among the four indicators of PC2, three indicators (SFW, TD and VD) after SP treatment ranked the top. This proved that SP had a positive influence on the appearance of the fruits. SP had the highest scores on PC3 and had the lowest TA and the highest SAR. This reflected that PC3 was positively related to SAR, while negatively related to TA. This also proved that ST had positive effects on the fruits’ taste. The above results were completely consistent with ANOVA results, indicating that simplification and recombination results based on PCA were reliable. On this basis, a comprehensive evaluation model of fruit quality indicators was built.

### 3.4 Comprehensive evaluation of fruit quality

Combining the contribution rates of principal components to the total variance in **Table 5**, a comprehensive evaluation model of fragrant pear was built:


$$f_{s}=\frac{t_{1}}{t_{1}+t_{2}+t_{3}}f_{1}+\frac{t_{2}}{t_{1}+t_{2}+t_{3}}f_{2}+\frac{t_{3}}{t_{1}+t_{2}+t_{3}}f_{3}$$

where *t_1_* ~*t_3_* refer to contribution rates of PC1, PC2 and PC3 to total variance. *f_s_* is the comprehensive score of fragrant pear fruit. *f_1_, f_2_*, and *f_3_* are scores of fragrant pear samples on PC1, PC2 and PC3. The numerical values of *f_1_, f_2_*, and *f_3_* were brought into the above formula. Then, the calculation formula of comprehensive evaluation scores of fragrant pears was gained:


$$f_{s}=0.36f_{1}+0.34f_{2}+0.3f_{3}$$

The comprehensive evaluation scores are listed in **Table 6**. The order of green manure planting modes in terms of comprehensive quality evaluation scores of fragrant pears was: ‘SA’>‘SP’>‘ST’>‘S’. PC1 of SA got the highest scores because four indicators of PC1 (VC, PRO, FH and SSC) were the highest among all four groups. However, SA achieved the lowest scores in three of four indicators of PC2 (SFW, SI and VD). Hence, the PC2 of SA ranked fourth. The PC1 scores of SP ranked second, which was only subsequent to SA. The PC2 of SP ranked at the top, and SI was relatively moderate. The SFW of SP ranked at the top, and it was far superior to the other three treatments because SAR, which reflects the flavour of fruits, was relatively low. The score of PC3 ranked fourth. The quality indicators in PC3 were optimal after ST treatment. Moreover, PC1 and PC2 were below the moderate level and ranked third. Among the fruit traits of PC1 in S, nutritive values of fruits (VC and PRO), post-harvest transportation and storage performances (FH) and fruit maturity (SSC) were relatively low. Hence, scores of PC1 were the lowest and ranked fourth. The score of PC2 and PC3 ranked second. The contained appearance indicators and flavour indicators were above the moderate level.

**Table 6:** Comprehensive evaluation scores of fruit quality.

Treatments	Factor 1 score	Sort	Factor 2 score	Sort	Factor 3 score	Sort	Overall score	Sort
SA	2.79	1	-1.50	4	-0.39	3	0.39	1
SP	0.05	2	1.40	1	-1.26	4	0.12	2
ST	-1.11	3	-0.35	3	1.61	1	-0.05	3
S	-1.72	4	0.45	2	0.04	2	-0.46	4

## 4 Discussion

In modern agricultural production, due to the lack of scientific understanding and guidance of chemical fertilizer, farmers blindly applied chemical fertilizer in pursuit of high yield of crops, resulting in the imbalance of soil nutrient structure, the deterioration of physical properties, and the decline of fertility. In addition, residues of some chemical substances such as nitrogen, phosphorus and potassium in chemical fertilizers continue to accumulate in the soil, resulting in nutrient imbalance in the soil, hindering the transformation and absorption of nutrients by crops, and resulting in the decline of agricultural product quality (Zhao et al., 2020; Xiao et al., 2022). Green manure practice is an extensive soil improvement strategy in organic agriculture (Verzeaux et al., 2016). Green manure crops contain a large amount of organic matter, which can improve soil structure and improve soil water and fertilizer retention capacity (Bohm et al., 2020; Gomez and Soriano, 2020). At the same time, organic acids produced by the secretion and decomposition of green manure crops during the growth process can transform the insoluble phosphorus and potassium in the soil into available elements, which is conducive to the absorption and utilization of crops, and further improve the quality of fruits (Arruda et al., 2021). The relationship between green manure cover crop cultivation and crop quality has been widely researched. Pisciotta et al. (2021) indicated that leguminous green fertilizer increased the soluble solid content of fruits, and this research also found that the application of leguminous green fertilizer increased the soluble solid content of Korla fragrant pear. This may be because the roots of leguminous cover crops are rich in nitrogen, which degrades faster in soil after turning, providing an important nitrogen source for the growth of fruit trees (Gaskell and Smith, 2007), and thus improving the intrinsic quality of fruit. However, some researches found that the Leguminosae green (*Trifolium squarrosum* L.) as a cover crop did not significantly improve the soluble solids and hardness of fruits (Abou Chehade et al., 2019), which was different from the results of this research. In addition, there are researches have shown that Leguminosae green fertilizer and nitrogen fertilizer have no significant effect on the weight per fruit of watermelon (Fracchiolla et al., 2019), which is consistent with the results of our paper. Poaceae green manure also plays a huge role in the sustainability of agricultural production (Bedoussac et al., 2015). Intercropping Poaceae green manure improved the community structure and biological characteristics of soil bacteria (Gong et al., 2019). Zhu et al. (2022) found that Poaceae green manure significantly increased the vitamin C content of fruits, which was consistent with our results. In our research, it was found that the fruit weight of green manure planting mode SP was the largest, with an average weight of 183.26g (**Table 3**). Bhat et al. (2014) compared the effects of Leguminosae green manure and Poaceae green manure on apple weight, and found that Leguminosae green manure significantly increased fruit weight and yield per fruit, while Poaceae green manure had no significant effect on fruit weight, which was different from the results of our research. Our research found that oilseed rape green manure significantly increased the sugar-acid ratio of Korla pear fruits, similarly, Wang et al. (2020) found that high concentration of oilseed rape green manure significantly increased the sugar content of fruits. In addition, it was found that oilseed rape had low requirements on the growing environment (drought tolerance, salt tolerance), and had significant effect on weed control, reducing the workload of weed control, and had a good prospect for popularization and application in arid areas.

There are more than 30 varieties of fragrant pear in Xinjiang, China, among which only Korla fragrant pear is cultivated and sold in a standardized model, which shows its extremely high commodity value (Zari et al., 2021). In recent years, consumers have become more interested in organic fruits, and Korla fragrant pear, which is grown with green cover crops and used as fertilizer, is an organic agricultural product, which is helpful to improve its commodity value. However, fruit quality is a determinant of economic value and market competitiveness of the pear industry (Wei et al., 2017). The market value of fragrant pear is closely related to the size, shape, texture, nutrition and flavor of the fruit. In addition, in the process of orchard management, cover crops between fruit tree rows will form a small ecological circle on the surface, which is conducive to maintaining water and promoting nutrient cycling (Yuarsah and Handayani, 2017), and also effectively inhibits the erosion of main crops by pests (Bowers et al., 2020; Beaumelle et al., 2021). Therefore, the screening of orchard cover crops should be based on fruit quality, and then combined with the actual requirements of agronomy to carry out evaluation, in order to determine the suitable cover crops for orchard, and better play the advantages of green manure.

## 5 Conclusion

The results showed that different planting modes of green manure had different effects on the quality of Korla pear. Compared with planting mode S (Orchard authigenic green manures), the soluble solid content (SSC), protein (PRO), vitamin C (VC), and fruit hardness (FH) in fruits of SA (Leguminosae green manures alfalfa) were improved by 9.57%, 13.64%, 44.03, and 30.97%, respectively, the single-fruit weigh (SFW) of SP (Poaceae green manures oats) was improved by 7.48%, the Sugar-acid ratio (SAR) of ST (Cruciferae green manures oilseed rape) was improved by 22.08%. The eleven quality indicators were divided into three principal components according to principal component analysis (PCA), and the contribution rate to total variance is 81.078%. These three principal components reflect internal quality (29.508%), appearance traits (27.624%) and tastes (23.946%), respectively. According to contribution rates of principal components, a comprehensive fruit quality evaluation model of fragrant pears under different green manures was built. For comprehensive scores, there was an order: ‘SA’>‘SP’>‘ST’>‘S’. Specifically, SA dramatically improves the internal quality and nutritive value of fruits. SP increases SFW of fruits, while ST markedly improves taste indicators of fruits.

## Data availability statement

The original contributions presented in the study are included in the article/supplementary material. Further inquiries can be directed to the corresponding author.

## Author contributions

Resources, JL; data curation, XL and YL; writing—original draft preparation, SH and JZ; writing—review and editing, YL and JZ; visualization, GS; supervision, JL; project administration, JL and LX. All authors have read and agreed to the published version of the manuscript.

## Funding

This is research was financially supported by the China Agriculture Research System of MOF and MARA (CARS-22), Xinjiang Construction Corps, grant number (2021CB022), the Finance science and technology project of Alar City (2021NY07), the Key neighborhood Science and Technology Project of Xinjiang Construction Corps (2018AB037), President’s Foundation Innovation Research Team Project of Tarim University(TDZKCX202203) and the Finance science and technology project of Alar City (2022NY13)

## Conflict of interest

The authors declare that the research was conducted in the absence of any commercial or financial relationships that could be construed as a potential conflict of interest.

## Publisher’s note

All claims expressed in this article are solely those of the authors and do not necessarily represent those of their affiliated organizations, or those of the publisher, the editors and the reviewers. Any product that may be evaluated in this article, or claim that may be made by its manufacturer, is not guaranteed or endorsed by the publisher.
